# Roles of ITPA and IL28B Genotypes in Chronic Hepatitis C Patients Treated with Peginterferon Plus Ribavirin 

**DOI:** 10.3390/v4081264

**Published:** 2012-08-14

**Authors:** Tatsuo Miyamura, Tatsuo Kanda, Shingo Nakamoto, Shuang Wu, Xia Jiang, Makoto Arai, Keiichi Fujiwara, Fumio Imazeki, Osamu Yokosuka

**Affiliations:** 1 Department of Medicine and Clinical Oncology, Graduate School of Medicine, Chiba University, 1-8-1 Inohana, Chuo-ku, Chiba 260-8677, Japan; Email: miyamura__ta@hotmail.com (T.M.); nakamotoer@yahoo.co.jp (S.N.); wushuang@graduate.chiba-u.jp (S.W.); jxia925@yahoo.co.jp (X.J.); araim-cib@umin.ac.jp (M.A.); fujiwara-cib@umin.ac.jp (K.F.); imazekif@faculty.chiba-u.jp (F.I.); yokosukao@faculty.chiba-u.jp (O.Y.); 2 Department of Molecular Virology, Graduate School of Medicine, Chiba University, 1-8-1 Inohana, Chuo-ku, Chiba 260-8677, Japan; Email: nakamotoer@yahoo.co.jp

**Keywords:** Anemia, HCV, IL28B, ITPA, SNP, sustained virological response

## Abstract

It has been reported that inosine triphosphatase (ITPA) gene variants protect against ribavirin-induced anemia in patients treated for chronic hepatitis C. IL28B variants also influence the treatment response of peginterferon plus ribavirin treatment in these patients. In the present study, we examined how ITPA and IL28B genotypes have clinical impacts on treatment-induced hematotoxicities and treatment response in HCV-infected patients treated with peginterferon plus ribavirin. ITPA genotypes (rs1127354 and rs6051702) and IL28B genotype (rs8099917) were determined by TaqMan SNP assay. We compared clinical background, treatment course and treatment response in terms of these genotypes. Only IL28B rs8099917 major type could predict sustained virological response. ITPA rs1127354 major type leads to significantly greater ribavirin-induced anemia than ITPA rs1127354 minor type between days 0 and 84. We noticed that IL28B rs8099917 minor genotype was associated with higher reduction of neutrophils and platelets. ITPA rs1127354 is useful for the prediction of ribavirin-induced anemia in the early phase after the commencement of peginterferon plus ribavirin treatment and IL28B rs8099917 is useful for the prediction of sustained virological response. Use of the combination of these two genotypes could lead to safe and effective treatment of chronic hepatitis C patients.

## 1. Introduction

Chronic hepatitis C virus (HCV) infection is a major cause of hepatocellular carcinoma (HCC) and a leading cause of end-stage liver disease worldwide [1]. The current standard therapy is based on a combination of peginterferon and ribavirin, but this treatment leads to only about 50% sustained virological response (SVR) in patients with HCV genotype 1 and high viral loads, who were mostly null-responders or relapsers [2]. Recently, the direct-acting antiviral (DAA) agents boceprevir and telaprevir were licensed for the treatment of HCV infection [3], and these drugs might be more powerful tools for HCV-infected patients.

Interleukin 28B (IL28B) variants influence the treatment response of peginterferon plus ribavirin treatment in HCV-infected patients [4,5,6,7,8]. Genome-wide association study has revealed a strong relationship between single-nucleotide polymorphisms (SNPs) near IL28B on chromosome 19 and null virological response in the treatment of patients with HCV genotype 1 in Australian [4], Japanese [5] and other populations [6]. Baseline plasma interferon-gamma inducible protein 10 kDa (IP-10 or CXCL10) is significantly associated with IL28B-related SNPs, and augments the level of predictiveness of the first-phase decline in HCV RNA, rapid virological response (RVR) and final treatment outcome [9,10]. Further studies will be needed to reveal the mechanism concerning IL28B and the response to interferon.

It has also been reported that inosine triphosphatase (ITPA) gene variants protect against ribavirin-induced hemolytic anemia in chronic hepatitis C patients [11]. Proposed mechanisms of action for ribavirin against HCV include (1) direct effect against HCV RNA-dependent RNA polymerase [12], (2) induction of misincorporation of nucleotides leading to lethal mutagenesis [13,14], (3) depletion of intracellular pools via inhibition of inosine monophosphate dehydrogenase [15], (4) alteration in the cytokine balance from a Th2 profile (anti-inflammatory) to a Th1 profile (pro-inflammatory) [16], and (5) potentiating the effect of interferon via up-regulation of genes involved in interferon signaling [17,18]. Clinical studies provide strong evidence for the benefit of ribavirin in combination with DAAs for both interferon containing and sparing regimens [18].

In the present study, we examined how ITPA and IL28B genotypes clinically contribute to treatment-induced hematotoxicities and treatment response in HCV-infected patients treated with peginterferon plus ribavirin. We found that IL28B rs8099917 minor genotype was associated with greater reduction of neutrophils and platelets. Use of a combination of these genotypes could lead to a safe and effective treatment for chronic hepatitis C patients. It is conceivable that these variants may modulate treatment responses as well as treatment pathways, and the result of this study might show the way of the future direction of these gene variants in treatment or drug development.

## 2. Results

### 2.1. Patient Characteristics According to IL28B and ITPA Genotypes

First, we genotyped IL28B rs8099917, and ITPA rs1127354 and rs6051702 in 97 HCV-infected patients (Table 1). Sixty and 37 patients possessed IL28B rs8099917 major and minor genotypes, respectively. Seventy-four and 23 patients possessed ITPA rs1127354 major and minor genotypes, respectively, and 59 and 38 possessed ITPA rs6051702 major and minor genotypes, respectively.

**Table 1:** Background of study population at enrollment.

Study variables	Total (n = 97)
Age (years)	55.1 ± 10.8
Gender (male/female)	44/53
***SNP genotype***	
*IL28B rs8099917* TT/TG/GG	60/35/2
*ITPA rs1127354* CC/CA/AA	74/21/2
*ITPA rs6051702* AA/AC/CC	59/32/6
***Response to previous therapy***	
Naïve/relapse/null response	67/17/13
HCV RNA (H/L)	95/2
HCV genotype (G1/G2)	81/16
AST (IU/L)	56.0 ± 49.4
ALT (IU/L)	67.9 ± 62.4
γGTP (IU/L)	53.5 ± 73.2
WBC (/mm^3^)	5,410 ± 1,640
Hemoglobin (g/dL)	14.0 ± 1.1
Platelets (×10^4^/mm^3^)	17.5 ± 5.1
History of diabetes mellitus (+/−)	15/82
US (CLD/cirrhosis/unknown)	83/12/2
***Treatment Response***	
RVR (+/−/unknown)	14/82/1
EVR (+/−)	52/45
SVR (+/relapser/null/unknown)	40/27/22/8

H, high viral load (≥5 log IU/mL); L, low viral load (<5 log IU/mL); G1, genotype 1; G2, genotype 2; WBC, white blood cell count; US, ultrasound finding; CLD, chronic liver disease.

IL28B rs8099917 major-type patients included more interferon treatment-naïve patients than minor-type patients. Lower γGTP levels were seen in IL28B rs8099917 major-type patients (Table 2). ITPA rs1127354 major-type patients were older than ITPA rs1127354 minor-type patients and tended to be female-dominant in the present study (Table 2).

### 2.2. Treatment Response According to IL28B and ITPA Genotypes

Next, we compared the treatment response among patients according to IL28B and ITPA genotypes (Table 3). IL28B rs8099917 could predict SVR, as previously reported [4,5,6,7,8,9], while both ITPA genotypes did not in the present study. We reconfirmed that IL28B rs8099917 is one of the predictive values for treatment response in interferon-included regimens.

**Table 2:** Baseline characteristics of patients grouped according to *IL28B* and *ITPA* genetic variations.

Study variables	*IL28B rs8099917*			*ITPA rs1127354*			*ITPA rs6051702*		
	TT	TG/GG	*P-value*	CC	CA/AA	*P-value*	AA	AC/CC	*P-value*
No. of patients	60	37		74	23		59	38	
Age (years)	55.7 ± 11.2	54.7 ± 10.1	N.S.	56.8 ± 9.7	49.6 ± 12.2	0.0043	55.6 ± 11.3	54.4 ± 9.9	N.S.
Gender (male/female)	25/35	19/18	N.S.	29/45	15/8	0.0511	29/30	15/23	N.S.
***Response to previous therapy*** (naïve/relapse/null response)	46/10/4	21/7/9	0.029	48/17/9	19/0/4	N.S.	40/12/7	27/5/6	N.S.
HCV RNA (H/L)	58/2	37/0	N.S.	73/1	22/1	N.S.	58/1	37/1	N.S.
HCV genotype (G1/G2)	49/11	32/5	N.S.	63/11	18/5	N.S.	48/11	33/5	N.S.
AST (IU/L)	53.3 ± 56.2	60.3 ± 36.0	N.S.	52.8 ± 31.9	66.2 ± 84.4	N.S.	51.6 ± 30.1	62.8 ± 69.5	N.S.
ALT (IU/L)	62.4 ± 65.3	76.9 ± 57.0	N.S.	62.3 ± 48.5	85.7 ± 93.5	N.S.	62.4 ± 47.5	76.4 ± 80.2	N.S.
γGTP (IU/L)	35.5 ± 34.5	82.8 ± 104	0.0016	55.1 ± 80.9	48.7 ± 40.1	N.S.	51.6 ± 72.1	56.5 ± 75.6	N.S.
WBC (/mm^3^)	5580 ± 1820	5140 ± 1260	N.S.	5390 ± 1630	5470 ± 1680	N.S.	5570 ± 1690	5160 ± 1540	N.S.
Hb (g/dL)	13.9 ± 1.1	14.3 ± 1.1	N.S.	13.9 ± 1.0	14.3 ± 1.2	N.S.	14.0 ± 1.1	14.0 ± 1.1	N.S.
Platelets (×10^4^/mm^3^)	17.9 ± 5.3	16.8 ± 5.0	N.S.	17.4 ± 5.4	17.7 ± 4.3	N.S.	17.8 ± 5.4	17.0 ± 4.7	N.S.
History of diabetes mellitus (+/−)	9/51	7/30	N.S.	11/63	4/19	N.S.	8/51	7/31	N.S.
US (CLD/cirrhosis/unknown)	51/8/1	32/4/1	N.S.	62/10/2	21/2	N.S.	50/8/1	33/4/1	N.S.

H, high viral load (≥5 log IU/mL); L, low viral load (<5 log IU/mL); G1, genotype 1; G2, genotype 2; WBC, white blood cell count; US, ultrasound finding; CLD, chronic liver disease.

**Table 3:** Treatment response in patients grouped according to *IL28B* and *ITPA* genetic variations.

Study variables	*IL28B rs8099917*			*ITPA rs1127354*			*ITPA rs6051702*		
	TT	TG/GG	*P-value*	CC	CA/AA	*P-value*	AA	AC/CC	*P-value*
No. of patients	60	37		74	23		59	38	
RVR (+/−/unknown)	12/47/1	2/35/0	0.085	10/63/1	4/19/0	N.S.	10/49/0	4/33/1	N.S.
EVR (+/−)	43/17	9/28	0.000014	36/38	16/17	N.S.	31/28	21/17	N.S.
SVR (+/Relapser/Null/unknown)	29/6/18/7	11/16/9/1	0.042	28/17/22/7	12/5/5/1	N.S.	27/13/16/3	13/9/11/5	N.S.

RVR, rapid virological response; EVR, early virological response; SVR, sustained virological response.

### 2.3. Ribavirin-Induced Anemia According to IL28B and ITPA Genotypes

**Figure 1:**
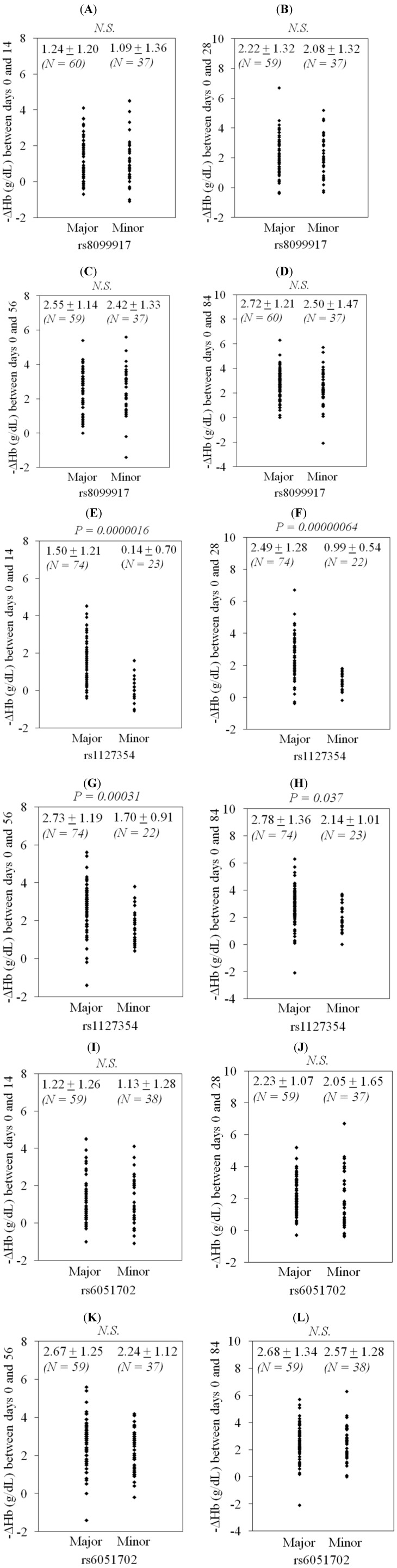
Ribavirin-induced reduction of hemoglobin according to IL28B and ITPA genotypes. (**A**)–(**D**), IL28B rs8099917; (**E**)–(**H**), ITPA rs1127354; (**I**)–(**L**), ITPA rs6051702. (**A**), (**E**) and (**I**) show the changes of hemoglobin (Hb) between days 0 and 14, (**B**), (**F**) and (**J**) between days 0 and 28, (**C**), (**G**) and (**K**) between days 0 and 54, and (**D**), (**H**) and (**L**) between days 0 and 84.

Next, we examined ribavirin-induced anemia among patients according to IL28B and ITPA genotypes (Figure 1). IL28B rs8099917 did not influence ribavirin-induced anemia (Figure 1A–D), nor did ITPA rs6051702 (Figure 1I-1L). ITPA rs1127354 major type led to significantly greater ribavirin-induced anemia than ITPA rs1127354 minor type in Japanese patients during peginterferon plus ribavirin treatment (Figure 1E-1H). 

### 2.4. Association Between ITPA rs1127354 Genotype and Dose Reduction of Drugs During Treatment

Next, we investigated the association between ITPA rs1127354 genotype and dose reduction of drugs at day 28 (Table 4). ITPA rs1127354 genotype could not predict the dose reduction of peginterferon (Table 4A), but ITPA rs1127354 major type could predict the dose reduction of ribavirin (Table 4B). We also examined the association between ITPA rs1127354 genotype and dose reduction of drugs at day 84 (data not shown). In patients with reduced ribavirin and/or peginterferon with null response, and in patients relapsed to the treatment, the proportion of patients with ITPA rs1127354 major type was greater among the patients with reduced ribavirin doses than among those with reduced peginterferon doses (20/20, 100% *vs.* 12/15, 80%; *P =* 0.036).

**Table 4:** Association between ITPA rs1127354 genotype and dose reduction of drugs at day 28. (A) Pegylated interferon (N = 74, no statistically significant difference); (B) Ribavirin (N = 74, *P* = 0.0071)

Study variables	*ITPA rs1127354* major type	*ITPA rs1127354* minor type
**A**		
Dose reduction (+)	17	4
Dose reduction (−)	57	19
**B**		
Dose reduction (+)	22	0
Dose reduction (−)	52	23

### 2.5. Effects of IL28B and ITPA Genotypes on the Reduction of White Blood Cell/Neutrophil Count

Next, we investigated the association between IL28B and ITPA genotypes, and other hematotoxicities between days 0 and 14, 28, 56 and 84 (data not shown). IL28B rs8099917 minor type induced higher reduction of white blood cell count (*P =* 0.043) as well as neutrophil count between days 0 and 14 (*P =* 0.034). We also analyzed the neutropenia, adjusting for background difference, and we confirmed these data. ITPA rs1127354 major type induced higher reduction of white blood cell count (*P =* 0.035) as well as higher reduction of neutrophil count between days 0 and 28 (*P =* 0.020). These genotypes had no effects on the reduction of white blood cell and neutrophil counts at any other time points, and ITPA rs6051702 had no effects on these reductions at any of the time points.

### 2.6. Effects of IL28B and ITPA Genotypes on the Reduction of Platelet Count

IL28B rs8099917 minor type induced higher reduction of platelet count between days 0 and 14 (*P =* 0.013) as well as between days 0 and 84 (*P =* 0.032) (data not shown). We also analyzed the thrombocytopenia, adjusting for the background difference, and we confirmed these data. ITPA rs1127354 minor-type induced higher reduction of platelet count between days 0 and 28 (*P =* 0.026) (data not shown). At any other time point these genotypes had no effects on the reduction of platelet count, and ITPA rs6051702 had no effects on this reduction at any time point.

## 3. Experimental Section

### 3.1. Patients

Between February 2010 and January 2011, blood samples were obtained from 97 chronic hepatitis C patients at the Department of Gastroenterology, Chiba University Medical School Hospital. Some of these patients had already been included in previous reports [7,8]. Written informed consent was obtained from each patient participating in this study. The study protocol conformed to the ethical guidelines of the Declaration of Helsinki and was approved by the ethics review committee of Chiba University, Graduate School of Medicine. Baseline characteristics are listed in Table 1. Sixty-seven and 30 patients were treatment-naïve and previously treated with interferon therapy, respectively. Previous relapse was defined as undetectable HCV RNA by the end of therapy [2], but then its reappearance after the end of therapy, and the definition of null response was less than 2 log_10_ decrease in HCV RNA from baseline after 12 weeks of therapy [2]. In 17 relapsers, 7, 3, 2, 2 and 3 received standard interferon monotherapy, standard interferon plus ribavirin, peginterferon monotherapy, peginterferon plus ribavirin and unknown, respectively. In 13 null-responders, 10, 1 and 2 received standard interferon monotherapy, standard interferon plus ribavirin and peginterferon plus ribavirin, respectively. Most patients were infected with HCV genotype 1 (83.5%) with high viral load (>5 log IU/mL) (97.9%). Ultrasound (US) findings showed cirrhosis of the liver in 12 cases (Table 1), 3 of which were also biopsy-proven.

### 3.2. Treatment

All 97 patients were treated with peginterferon-alfa once weekly and 400–1,000 mg of ribavirin daily [19,20,21]. Some of them stopped treatment at 12–16 weeks according to the early stopping rule.

### 3.3. HCV RNA Quantification

HCV RNA was determined by Amplicor HCV monitor assay, version 2.0 (range: 0.5–850 KIU/mL) (Roche Diagnostics, Tokyo, Japan), Amplicor HCV assay (Roche) or COBAS TaqMan HCV test (Roche) (range: 1.2–7.8 log IU/mL). The detection limit of this qualitative assay was 50 IU/mL, corresponding to 1.7 log IU/mL by COBAS TaqMan PCR assay [19]. We defined HCV RNA >5 log IU/mL and <5 log IU/mL as high and low viral titers of HCV RNA, respectively.

### 3.4. HCV Genotyping

HCV genotype was determined using the antibody-serotyping assay of Tsukiyama-Kohara *et al.* [22]. In this assay, HCV serotypes 1 and 2 correspond to genotypes 1a/1b and 2a/2b, respectively, according to Simmonds’ classification [23].

### 3.5. Classification of Treatment Outcome

Patients were classified as having achieved RVR and early virological response (EVR) if HCV RNA was undetectable (<50 IU/mL) in serum at treatment week 4 and week 12, respectively, and as having SVR if HCV RNA was undetectable in serum 24 weeks after the completion of therapy.

### 3.6. DNA Extraction and TaqMan SNP Assay

To prepare the DNA sample from blood cells, we used DNA Extract All Lysis Reagents (Applied Biosystems Inc., Foster City, CA, USA). A specific TaqMan genotyping assay was performed for rs1127354, rs6051702 and rs8099917. Primers were manufactured by Applied Biosystems. Thermal cycling was performed with the ABI Step One real-time PCR system according to the manufacturer’s protocol. Activation of TaqMan GTXpress Master Mix (Applied Biosystems) and the initial denaturation cycle was at 95 °C for 20 seconds, followed by 40 cycles at 95 °C for 3 seconds and 60 °C for 20 seconds. We analyzed IL28B rs8099917 TT as major type and TG/GG as minor type, ITPA rs1127354 CC as major type and CA/AA as minor type, and ITPA rs6051702 AA as major type and AC/CC as minor type in the present study.

### 3.7. Statistical Analysis

Data were expressed as mean ± standard deviation. We used univariate analyses to compare patient characteristics and outcomes, applying Student’s t-test or Chi-square test as appropriate. *P* < 0.05 was considered statistically significant.

## 4. Discussion and Conclusion

In the present study, we also observed that IL28B rs8099917 major genotype was useful for the prediction of treatment response, as in previous studies [4,5,6,7,8,9], which reported the association between IL28B genotypes and HCV eradication with peginterferon plus ribavirin therapies in chronic hepatitis C patients. SVR was strongly associated with IL28B major genotype (rs8099917 TT). Serum γGTP levels were significantly higher in IL28B rs8099917 minor-type patients, as we reported previously [8].

Previous studies [21,24] showed that HCV-infected patients who can be maintained on >80% of peginterferon and ribavirin dosage for the duration of treatment exhibit enhanced SVR rates. Adherence to therapy decreased over time with both antiviral medications, but more so with ribavirin [25]. Ribavirin could be associated with clinically significant hemolytic anemia, resulting in its necessary dose reduction or discontinuation [26,27]. However, we did not observe any association between ITPA genotypes and SVR.

We also observed that ribavirin-induced anemia is highly dependent on the ITPA rs1127354 genotypes between days 0 and 84, and ITPA rs1127354 major type has been reported to be associated with a reduction in hemoglobin between weeks 0 and 4 [28,29]. In the present study, we observed a difference in age between ITPA rs1127354 major and minor types (Table 2), albeit with a rather limited number of the latter patients. In this respect, further study will be needed, although our previous study showed that the SVR rate of patients aged ≤65 years was similar to that of patients aged >65 years [21]. Genetic variation of ITPA causing an accumulation of inosine triphosphate (ITP) could result in ribavirin-induced anemia. ITP confers protection against ribavirin-induced adenosine triphosphate (ATP) reduction by substituting for erythrocyte GTP, which is depleted by ribavirin, in the biosynthesis of ATP [30]. It is possible that ribavirin-induced anemia is due primarily to the effect of the drug on GTP and consequently ATP levels in erythrocytes [30].

Interestingly, we found that IL28B rs8099917 minor genotype was associated with greater reductions of neutrophils and platelets, although it was reported that IL28B polymorphisms were not associated with interferon-related cytopenia [31]. Our data support the previous reports that patients with ITPA rs1127354 major type had a higher degree of reactive increase in platelet count [32,33]. Further studies will be needed to investigate the potential underlying mechanism and to examine whether there is a synergistic effect of IL28B and ITPA. In the not-too-distant future, HCV therapy will likely move away from interferon-based regimens with increasing numbers of potent antiviral agents being approved, meaning that IL28B and/or ITPA genotyping would not play any additional role and be useful in clinical practice [34,35,36].

Recent studies revealed that IL28B is associated with hepatic interferon-stimulated gene (ISG) expression [10], hepatic STAT1-nuclear localization [9], hepatic suppressor of cytokine signal 3 (SOCS3) [37] and plasma interferon-gamma inducible protein-10 (IP-10) levels in chronic HCV infection [8]. It is possible that IL28B genotypes affect virus-host interaction through the interaction with interferon signaling pathways. IL28B major type also reported to be associated with a lower prevalence of hepatic steatosis and a less pronounced lipid metabolism, as reflected both by serum lipoprotein levels and hepatic steatosis in HCV infection [38,39,40,41]. Insulin resistance is more common in IL28B minor genotype than in major type in treatment-naïve patients with chronic hepatitis C [42,43]. Although there are contrary opinions [44,45], IL28B genotypes influence the stage of liver fibrosis [46,47] and HCV-related hepatocarcinogenesis [48]. Thus, IL28B genotypes play important roles in not only eradication of HCV but also HCV-related pathology.

In HCV infection, patients who developed HCC had lower platelet counts [49]. It is well known that the platelet count decreased with stage advancement of liver diseases in chronic hepatitis C patients [2,49,50,51,52]. Chronic hepatitis C is associated with variable degrees of anemia, neutropenia, and/or thrombocytopenia [52]. Multiple factors, including ITPA genotypes, might be involved in this phenomenon.

Our study showed that about 60% of Japanese patients infected with HCV have the preferable allele of IL28B rs8099917, but about 70% of patients also have the undesirable allele of ITPA rs1127354. There seem different distributions between IL28B and ITPA genotypes in the world [6,11]. In conclusion, ITPA rs1127354 is useful for the prediction of ribavirin-induced anemia in the earlier phase of peginterferon plus ribavirin treatment, and IL28B rs8099917 is useful for the prediction of SVR. Use of a combination of these genotypes could lead to a safe and effective treatment for chronic hepatitis C patients.
